# The relationship and interaction between triglyceride glucose index and obesity in the risk of prehypertension population: a cross-sectional study from a survey in Anhui, Eastern China

**DOI:** 10.1186/s12872-023-03365-x

**Published:** 2023-07-01

**Authors:** Jiaye Zhang, Linlin Jia, Tongying Zhu, Hao Zhu, Li Shu

**Affiliations:** School of Public Health, Bengbu Medical College, 2600 Donghai Road, Bengbu, 233030 Anhui Province China

**Keywords:** PHT, TyG index, Interaction, Obesity

## Abstract

**Background:**

The triglyceride glucose (TyG) index has been regarded as an effective proxy of Insulin resistance (IR). Studies on the TyG index, obesity and the risk of prehypertension (PHT) in elderly people are not apparent currently. The study sought to investigate the predictive value of TyG index and the associations with PHT risk and obesity.

**Methods:**

A community-based cross-sectional study was conducted in Bengbu City, Anhui province, China. Participants older than 65 years accepted questionnaire surveys, physical examinations and blood biochemistry tests. Based on the testing results, indicators including BMI (body mass index), WC (waist circumference), WHtR (waist-to-height-ratio), LAP(Lipid accumulation products) and TyG were calculated. Residents were classified into quartiles by their TyG indexes. Receiver operating characteristic curve (ROC) analysis was carried out to predict obesity indices for PHT. The three additive interaction indicators, RERI (relative excess risk due to interaction), AP (attributable proportion due to interaction) and S (synergy index) were used to assess the interaction impacts.

**Results:**

Two thousand six hundred sixty-six eligible elderly people were included in study and the prevalence of PHT was 71.04% (*n* = 1894). With increasing TyG index quartile, PHT became more prevalent. After adjusting for confounding factors, the prevalence of PHT risk with TyG levels in the fourth quartile (Q4, male: 2.83, 95%CI: 1.77–4.54; female: 2.75, 95%CI:1.91–3.97) was greater than that in the first quartile (Q1:ref). TyG index (AUC: 0.626, 95%CI: 0.602 to 0.650) was superior than BMI (AUC: 0.609, 95%CI: 0.584 to 0.633) in predicting PHT among females. Eventually, there were significant interactions of TyG index with obesity in males (General obesity: AP = 0.87, 95%CI: 0.72 to 1.02, S = 10.48, 95%CI: 3.43 to 31.97; Abdominal obesity: AP = 0.60,95%CI: 0.38 to 0.83, S = 3.53, 95%CI: 1.99 to 6.26) and females (General obesity: AP = 0.89, 95%CI: 0.79 to 0.98, S = 12.46, 95%CI: 5.61 to 27.69; Abdominal obesity: AP = 0.66, 95%CI: 0.51 to 0.82, S = 3.89, 95%CI:2.54 to 5.98).

**Conclusion:**

TyG index and PHT risk are tightly correlated. The risk of chronic disease in the elderly can be decreased by early detection of PHT utilizing the TyG index. In this research, the TyG index was more predictable than other indicators of obesity.

## Background

Hypertension (HTN), which as a typical chronic disease, is a significant risk factor for diseases such as kidney disease, microvascular problems and cardiovascular disease (CVD) [1]. HTN has gradually become one of the most serious public health problems recognized worldwide [2]. The risk of HTN is rising annually as a result of the aging population's increasing severity in recent years. According to the most recent epidemiology data, HTN affects more than 1.2 billion people worldwide [3]. In 2025, this number is predicted to increase to 1.56 billion [4]. HTN places a heavy strain on society and nations in terms of both financial and medical costs and it disproportionately affects residents of developing and low-income nations [5]. To lessen the likelihood of developing HTN, early identification of HTN risk factors and active intervention of those factors are crucial. The Seventh National Joint Committee of HTN (JNC 7) classified prehypertension (PHT) as a form of HTN in 2003 [6]. Research have indicated that those with PHT are much more prone than people with normal blood pressure to develop HTN and CVD [7, 8]. Therefore, people with PHT can be screened by blood pressure in the clinical setting. Early intervention is used to reduce the risk of PHT in the elderly and reduce the mortality rate of chronic diseases in people with HTN. Furthermore, numerous investigations [9, 10] have shown that the risk of PHT is associated with obesity and insulin resistance (IR). Obese individuals have a greater tendency to develop PHT.

The triglyceride glucose (TyG) index was a relatively simple and reliable alternative indicator for IR proposed by Simental-Mendia [11] in 2008. The TyG index has been extensively employed in the investigation of endocrine and CVD disorders in recent years. The correlation and predictive ability of TyG index with these diseases were confirmed by Zou Su [12], Li Minghui [13] and Zhang F [14].

The synergistic effect of HTN and diabetes on the risk of PHT needs to be taken considered. This cross-sectional study was carried out among the elderly non-diabetic and non-hypertensive population, to eliminate these confounding impacts. The main objective of this study was to explore the relationship between TyG index and the risk of PHT in the elderly population in Anhui Province, China. The second purpose was to compare the predictive ability of obesity indicators such as TyG index, LAP index, BMI, WC and WHtR on the risk of PHT in the elderly population. The final objective was to investigate any potential interaction between the TyG index and obesity on PHT risk.

## Methods

### Study population

**Fig. 1:**
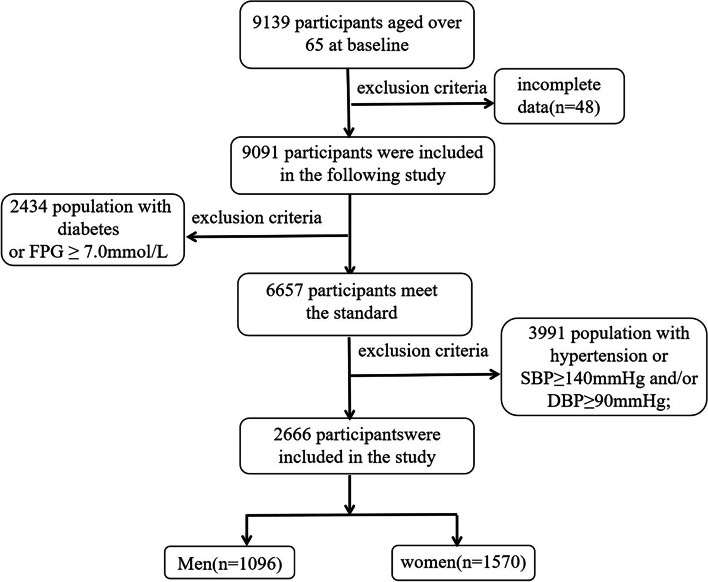
Flowchart of the selected participants in this study

This cross-sectional study called “Community-based Cardiovascular and Health Promotion Study” (COCHPS) was conducted in Bengbu City, Anhui Province, China. According to the results of a survey on HTN in China published in 2018 [15], the prevalence of PHT among people older than 65 years was about 30.5%. The allowable error is 3% and the confidence level was 1-ɑ = 0.95. PASS 11 software was used to calculate the sample size to be investigated as 936. Assuming that the non-response rate of the subjects was 10%, the sample size was required 1040 cases. Assuming that the qualified rate of the questionnaire is 90%, the total sample size was about 1156 cases. Finally,9139 community residents older than 65 years were selected in our study by a multistage random sampling. The inclusion criteria for this study were as follows: ①The participants did not have any mental illness or cognitive communication disorder; ②Participants lived in the communities for at least six months; ③Agreed to participate in the investigation; ④Elderly population. Exclusion criteria:①Unable or unwilling to complete the whole investigation; ②Residents with mental disorders; ③Subjects with diabetes or are taking hypoglycemic medications; ④Subjects with HTN or taking medication for high blood pressure. The data were screened in accordance with the requirements of the research. Figure 1 illustrates the eventual inclusion of 2666 participants. This study complied with the Declaration of Helsinki and had been approved by the Ethics Committee of Bengbu Medical College (No. BBMC-H-2021–098). All participants in the study provided written informed consent.

### Data collection

#### Questionnaire survey

A face-to-face questionnaire survey was conducted by qualified staffs. The following information were included in the questionnaire: ① Personal general information: name, sex, age, educational level (illiteracy/functionally illiterate, primary school, junior high school, high school graduate or higher), marital status (unmarried, married, single status); ② Life behaviors: current smoking (smoking continuously for more than a year and still smoking at least one cigarette each day in the past year), current drinking (drinking continuously for more than a year and still drinking 100 ml/d average in the past year), exercising (exercise for at least 20 min four or even more times a week is deemed adequate; otherwise, it is inadequate.) [16]; ③ dietary preference (meat preference, balanced diet, vegetarian preference): A 24-h recall method was used to assess the dietary preference, which was based on eight diverse food groups [17] (starchy staples, vitamin A rich vegetables and fruits, other vegetables and fruits, meat and poultry, fish and shrimp, eggs, legumes and nuts, milk and milk products). The dietary preference was counted the number of food groups that a resident consumed in the past 24 h. Any individual food item in each food group consumed by a resident earns one point for dietary preference, the meat preference was defined as more than three points intake of animal food, vegetarian preference was defined as more than three points intake of vegetable food, meanwhile, the balanced diet was considered as an equal amount intake of animal food and vegetable; ④Past medical history: HTN, diabetes and other medication histories.

#### Anthropometric measurements

An automatic height and weight measuring instrument (Heng Ding Technology DMH-301) was utilized by trained investigators to detect the height (cm) and weight (kg) of the research subjects. Participants were instructed to take their shoes and weights off and wear loose clothing. To determine the waist circumference of participants, the soft ruler was wrapped around a circle across the navel and measured at the end of exhalation. The measurement of waist circumference is in cm. Two decimal places were retained for these data.

#### Blood pressure measurement

Blood pressure was measured by an electronic sphygmomanometer (Omron HBP-1300) with an accuracy of 1 mmHg. To guarantee the accuracy of the value, participants should rest and sit for at least 10 min. The average of three times with blood pressure readings was measured for data analysis.

#### Laboratory examinations

Early in the morning, fasting blood samples (fasting for at least 8 h) were taken by medical practitioners. The measurement of fasting plasma glucose (FPG), triglycerides (TG), total cholesterol (TC), high-density lipoprotein cholesterol (HDL-C), low-density lipoprotein cholesterol (LDL-C) and other indicators was performed using an automatic biochemical analyzer (Modular P800, Switzerland).

### Definitions

#### The definitions of related chronic diseases

HTN: Systolic blood pressure (SBP) ≥ 140 mmHg and/or Diastolic blood pressure (DBP) ≥ 90 mmHg [6]; PHT: SBP 120-139 mmHg and/or DBP:80–89 mmHg [6]; diabetes: fasting plasma glucose (FPG) ≥ 7.0 mmol/L [16]; impaired fasting glucose(IFG): 6.1 ≤ FPG < 7 mmol/L [16]; hypercholesterolemia(HC):TC ≥ 6.22 mmol/L [18]. Metabolic syndrome (MetS): based on abdominal obesity (WC of males ≥ 90 cm, WC of females ≥ 80 cm) and combined two or more of the following(① TG ≥ 1.7 mmol/L; ② HDL-C of male < 1.03 mmol/L or HDL-C of female < 1.29 mmol/L; ③ SBP ≥ 130 mmHg or DBP ≥ 85 mmHg; ④ FPG ≥ 5.6 mmol/L) [19].

#### The definitions of the obesity index

General obesity was diagnosed with BMI, which calculated by weight (kg)/height (m^2^). lean: BMI < 18.5; normal: 18.5 ≤ BMI ≤ 23.9; overweight: BMI ≥ 24; obesity: BMI ≥ 28 [20]. Abdominal obesity was diagnosed with WC and WHtR. WC of males ≥ 90 cm or WC of females ≥ 85 cm [21]; WHtR = WC (cm)/height (cm); WHtR ≥ 0.5 was defined as abdominal obesity [21].

#### The definitions of the prediction index

The definitions of LAP were [WC (cm) -65] × [TG (mmol/L)] in males and [WC (cm) -58] × [TG (mmol/L)] in females [22]. The definition of TyG index is ln[TG(mg/dl) × FPG(mg/dl)/2] [11].

### Statistical analysis

PASS 15.0 (NCSS, LLC, Kaysville, Utah, USA), IBM SPSS Statistics version 25 (IBM SPSS Inc., Chicago, USA), R 4.1.2 (R Foundation for Statistical Computing, Vienna, Austria), MedCalc Version 18 (DEMO) software (MedCalc Software Ltd., Ostend, Belgium) and GraphPad Prism version 8.0.0 for Windows (GraphPad Software, San Diego, California USA) were used for statistical analysis of the survey data. The Kolmogorov–Smirnov test was utilized in this study to examine the normality of the data. The findings demonstrated that the normal distribution was not supported by *P* < 0.05. Kruskal–Wallis H Test and Mann–Whitney U test were employed to examine group differences, and continuity variables were specified by median and quartile spacing. Classification factors were presented as percentages of frequency (%), and the chi-square test was used to investigate differences across groups. For data analysis, the TyG index was separated into four groups according to quartiles (Q1, Q2, Q3, Q4). Multivariate logistic regression was used to study the relationship between PHT risk and TyG index in each group.

After adjusting confounding factors, Model 1 (Education level, Marital status, Physical activity, Dietary Preference, Current smoking, Current drinking were adjusted for the unadjusted model), Model 2 (The adjustment of IFG and HC were added on the basis of Model 1.) and Model 3 (The adjustment of FPG, TC, HDL and LDL were added on the basis of Model 2) were formed. ROC curve analysis was performed, sensitivity, specificity and Youden index were used to determine the differential ability of each index for PHT. By comparing the area under the curve (AUC), the prediction effect of different indicators on PHT risk was evaluated. Z test was used to test and analyze AUCs, and *p* values < 0.05, indicating statistical significance. Finally, the additive interaction indexes RERI, AP and SI were used to evaluate any potential additive interactions between obesity and the TyG index on PHT. All analyses were two-sided *P* value and *P* < 0.05 was considered statistically significant.

## Results

### Demographic and biochemical index of the study population

The profiles of participants with PHT and those with normal blood pressure are shown in Table 1. 2666 elderly subjects (1096 men and 1570 women) over the age of 65 years old met the criteria and included in this analysis. The prevalence of PHT was 837 (76.37*%*) in men and 1057 (67.32*%*) in women. In comparison to people with normotension, PHT participants exhibited significantly increased in weight, WC, TG, FPG, TyG index and LAP (*P* < 0.05). People with PHT or normotension had clearly differed from each other in terms of BMI level and the prevalence of metabolic syndrome (*P* < 0.05). Only among women were significant variations in dietary preference discovered (*P* < 0.05).

**Table 1:** Basic characteristics of blood pressure in men and women

Variables	Men		*Z/χ*^*2*^	*P*	Women		*Z/χ*^*2*^	*P*
	Normotension	Prehypertension			Normotension	Prehypertension		
	(*n* = 259)	(*n* = 837)			(*n* = 513)	(*n* = 1057)		
Age(years),M(P25,P75)	71(68,77)	71(67,76)	-0.307	0.759	70(67,75)	70(67,76)	-1.835	0.067
Age,n(*%*)			0.085	0.958			1.747	0.418
65–74	177(68.34)	568(67.66)			379(73.88)	749(70.86)		
75–89	80(30.89)	261(31.18)			129(25.15)	299(28.29)		
≥ 90	2(0.77)	8(0.96)			5(0.97)	9(0.85)		
Education level,n(*%*)			5.495	0.139			7.288	0.063
Illiteracy/Functionally illiterate	44(16.99)	125(14.93)			131(25.54)	235(22.23)		
Primary school	27(10.42)	131(15.65)			64(12.48)	183(17.31)		
Junior high school	142(54.83)	458(54.72)			258(50.29)	529(50.05)		
High school graduate or higher	46(17.76)	123(14.70)			60(11.70)	110(10.41)		
Marital status,n(*%*)			3.905	0.142			5.170	0.075
Unmarried	7(2.70)	30(3.58)			6(1.17)	30(2.84)		
Married	240(92.66)	787(94.03)			443(86.35)	914(86.47)		
Single status	12(4.63)	20(2.39)			64(12.48)	113(10.69)		
Dietary Preference,n(*%*)			2.000	0.368			17.758	0.000
Meat preference	4(1.54)	12(1.43)			4(0.78)	13(1.23)		
Balanced diet	211(81.47)	712(81.47)			386(75.24)	882(83.44)		
Vegetarian preference	44(16.99)	113(16.99)			123(23.98)	162(15.33)		
Physical activity,n(*%*)			0.187	0.666			0.494	0.482
Inadequate	158(61.00)	498(61.00)			309(60.23)	617(58.37)		
Adequate	101(39.00)	339(39.00)			204(39.77)	440(41.63)		
BMI,n(*%*)			25.809	0.000			42.771	0.000
< 18.5	12(4.63)	18(4.63)			29(5.65)	27(2.55)		
18.5–23.9	168(64.86)	427(64.86)			295(57.5)	470(44.47)		
≥ 24	72(27.80)	329(27.80)			161(31.38)	445(42.10)		
≥ 28	7(2.70)	63(2.70)			28(5.46)	115(10.88)		
Current smoking,n(*%*)	55(21.24)	150(21.24)	1.429	0.232	7(1.36)	14(1.32)	0.004	0.948
Current drinking,n(*%*)	60(23.17)	195(23.17)	0.002	0.965	18(3.51)	34(3.22)	0.092	0.762
Abdominal obesity,n(*%*)	67(25.87)	250(25.87)	1.539	0.215	310(60.43)	686(64.90)	2.978	0.084
IFG,n(*%*)	13(5.02)	53(5.02)	0.602	0.438	14(2.73)	71(6.72)	10.727	0.001
HC,n(*%*)	10(3.86)	45(3.86)	0.953	0.329	56(10.92)	148(14.00)	2.909	0.088
MetS,n(*%*)	10(3.86)	109(3.86)	17.153	0.000	38(7.41)	206(19.49)	38.407	0.000
Height(cm),M(P25,P75)	168(163,172)	169(165,172)	-1.655	0.098	156(152.5,160)	157(153.5,160)	-1.757	0.079
Weight(kg),M(P25,P75)	63(58.90,69.10)	68(62.95,73.55)	-6.702	0.000	55.9(51,61)	60(54,65)	-7.246	0.000
WC(cm),M(P25,P75)	83(79,90)	85(80.0,90.0)	-3.070	0.002	81(75,86)	82(76,88)	-2.301	0.021
BMI(kg/m2),M(P25,P75)	22.66(21.01,24.39)	23.82(22.22,25.71)	-6.470	0.000	22.94(21.24,24.87)	24.17(22.04,26.25)	-7.010	0.000
SBP(mmHg),M(P25,P75)	113(108.5,117)	128(123.5,132)	-23.326	0.000	113(107.5,117.5)	128(124,132)	-30.985	0.000
DBP(mmHg),M(P25,P75)	70(65,75)	79(74,82)	-14.805	0.000	70(65,75)	78(72.75,81)	-18.824	0.000
FPG(mmol/L),M(P25,P75)	4.83(4.42,5.30)	4.96(4.50,5.43)	-2.147	0.032	4.86(4.5,5.27)	4.99(4.59,5.45)	-4.346	0.000
TG(mmol/L),M(P25,P75)	0.90(0.67,1.20)	1.08(0.80,1.56)	-5.372	0.000	1.03(0.8,1.46)	1.3(0.92,1.74)	-7.380	0.000
TC(mmol/L),M(P25,P75)	4.40(3.90,5.10)	4.60(4.01,5.20)	-2.131	0.033	5.00(4.31,5.71)	5.1(4.47,5.70)	-2.165	0.030
LDL-C(mmol/L),M(P25,P75)	2.70(2.10,3.26)	2.79(2.29,3.30)	-1.727	0.084	3.00(2.27,3.57)	3.08(2.47,3.74)	-2.202	0.028
HDL-C(mmol/L),M(P25,P75)	1.21(0.99,1.53)	1.23(0.99,1.55)	-0.287	0.774	1.25(1.05,1.62)	1.32(1.06,1.67)	-1.464	0.143
TyG index,M(P25,P75)	8.12(7.82,8.49)	8.35(8.03,8.73)	-5.538	0.000	8.31(8.02,8.62)	8.57(8.21,8.89)	-8.125	0.000
LAP,M(P25,P75)	15.60(15.60,24.18)	21.06(13.16,34.00)	-5.401	0.000	24.00(15.25,36)	30.40(19.44,47.04)	-6.250	0.000
WHtR,M(P25,P75)	0.49(0.47,0.53)	0.50(0.48,0.54)	-2.409	0.016	0.52(0.48,0.56)	0.52(0.48,0.56)	-1.732	0.083

### Characteristics of the study population by TyG index quartiles

**Fig. 2:**
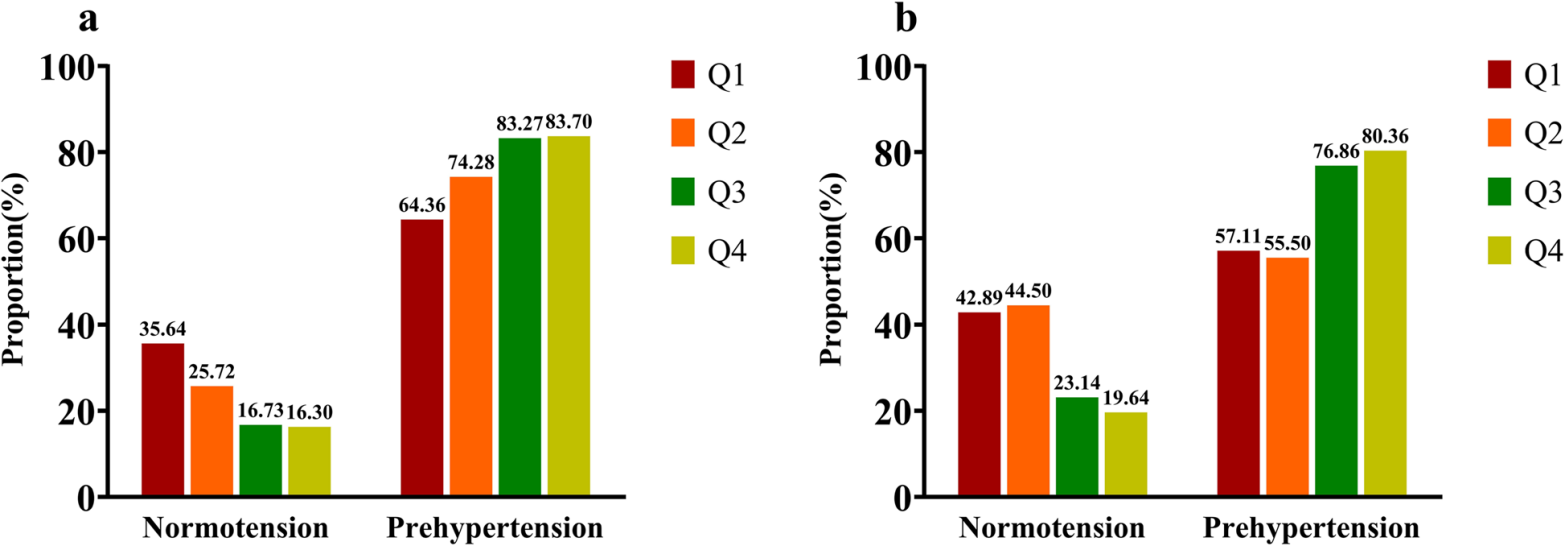
Prevalence of normotension and prehypertension in TyG index quartiles of different gender. **a** Prevalence of prehypertension in male TyG index quartiles. **b** Prevalence of prehypertension in female TyG index quartiles

Figure 2 demonstrated that as the TyG index quartiles increased, so did the prevalence of PHT in general, with an upward tendency. The clinical characteristics of the study population according to the quartiles of the TyG index were shown in Table 2. Significant differences were discovered among the TyG index quartile groups of subjects for LDL-C, HDL-C, current drinking, abdominal obesity, BMI levels, blood pressure status, prevalence of IFG, and MetS (*P* < 0.05). WC, BMI, FPG, TG, TC and LAP rose progressively across the quartiles of the TyG index (all *P* < 0.05). In addition, the age of men and the education levels and dietary preference of women were also observed significant differences in TyG index quartiles (*P* < 0.05).

**Table 2:** The baseline characteristics of subjects according to the quartile of TyG index

Variables	Men				*H/χ*^*2*^	*P*	Women				*H/χ*^*2*^	*P*
	Q1	Q2	Q3	Q4			Q1	Q2	Q3	Q4		
	(< 7.97)	(7.97–8.29)	(8.29–8.71)	(> 8.71)			(< 8.12)	(8.12–8.48)	(8.48–8.82)	(> 8.82)		
n	275	276	275	270			394	400	389	387		
Age(years),M(P25,P75)	73(68,77)	72(68,79)	70(67,75)	69(67,74)	29.571	0.000	70(67,75)	70.5(67,76)	70(67,75)	70(67,75)	2.473	0.480
Age,n(*%*)					31.258	0.000					4.892	0.558
60–74	172(62.55)	163(59.06)	199(72.36)	211(78.15)			286(72.59)	275(68.75)	286(73.52)	281(72.61)		
75–89	100(36.36)	111(40.22)	72(26.18)	58(21.48)			104(26.40)	120(30.00)	99(25.45)	105(27.13)		
≥ 90	3(1.09)	2(0.72)	4(1.45)	1(0.37)			4(1.02)	5(1.25)	4(1.03)	1(0.26)		
Education level,n(*%*)					10.851	0.286					29.253	0.001
Illiteracy/Functionally illiterate	52(18.91)	45(16.30)	37(13.45)	35(12.96)			125(31.73)	98(24.50)	75(19.28)	68(17.57)		
Primary school	46(16.73)	30(10.87)	43(15.64)	39(14.44)			55(13.96)	69(17.25)	59(15.17)	64(16.54)		
Junior high school	136(49.45)	161(58.33)	154(56.00)	149(55.19)			171(43.40)	194(48.50)	210(53.98)	212(54.78)		
High school graduate or higher	41(14.91)	40(14.49)	41(14.91)	47(17.41)			43(10.91)	39(9.75)	45(11.57)	43(11.11)		
Marital status,n(*%*)					10.076	0.121					7.000	0.321
Unmarried	15(5.45)	8(2.90)	5(1.82)	9(3.33)			9(2.28)	6(1.50)	9(2.31)	12(3.10)		
Married	248(90.18)	263(95.29)	264(96.00)	252(93.33)			330(83.76)	353(88.25)	335(86.12)	339(87.60)		
Single status	12(4.36)	5(1.81)	6(2.18)	9(3.33)			55(13.96)	41(10.25)	45(11.57)	36(9.30)		
Dietary Preference,n(*%*)					11.688	0.069					27.900	0.000
Meat diet	3(1.09)	4(1.45)	3(1.09)	6(2.22)			3(0.76)	3(0.75)	7(1.80)	4(1.03)		
Balanced diet	219(79.64)	242(87.68)	229(83.27)	233(86.30)			293(74.37)	320(80.00)	315(80.98)	340(87.86)		
Vegetarian diet	53(19.27)	30(10.87)	43(15.64)	31(11.48)			98(24.87)	77(19.25)	67(17.22)	43(11.11)		
Physical activity,n(*%*)					2.496	0.476					0.409	0.938
Inadequate	172(62.55)	170(61.59)	156(56.73)	158(58.52)			236(59.9)	233(58.25)	232(59.64)	225(58.14)		
Adequate	103(37.45)	106(38.41)	119(43.27)	112(41.48)			158(40.10)	167(41.75)	157(40.36)	162(41.86)		
BMI,n(*%*)					56.121	0.000					77.173	0.000
< 18.5	15(5.45)	10(3.62)	3(1.09)	2(0.74)			31(7.87)	15(3.75)	7(1.80)	3(0.78)		
18.5–23.9	175(63.64)	163(59.06)	137(49.82)	120(44.44)			220(55.84)	215(53.75)	182(46.79)	148(38.24)		
≥ 24	80(29.09)	87(31.52)	115(41.82)	119(44.07)			117(29.70)	138(34.50)	157(40.36)	194(50.13)		
≥ 28	5(1.82)	16(5.80)	20(7.27)	29(10.74)			26(6.60)	32(8.00)	43(11.05)	42(10.85)		
Blood pressure status,n(*%*)					37.944	0.000					90.115	0.000
Normotension	98(35.64)	71(25.72)	46(16.73)	44(16.30)			169(42.89)	178(44.50)	90(23.14)	76(19.64)		
Prehypertension	177(64.36)	205(74.28)	229(83.27)	226(83.70)			225(57.11)	222(55.50)	299(76.86)	311(80.36)		
Current smoking,n(*%*)	59(25.09)	45(16.30)	52(18.91)	49(18.15)	2.476	0.480	6(1.52)	2(0.50)	4(1.03)	9(2.33)	5.373	0.146
Current drinking,n(*%*)	69(25.09)	52(18.84)	80(29.09)	54(20.00)	10.380	0.016	14(3.55)	15(3.75)	14(3.60)	9(2.33)	1.587	0.662
Abdominal obesity,n(*%*)	48(17.45)	71(25.72)	94(34.18)	104(38.52)	34.760	0.000	204(51.78)	259(64.75)	251(64.52)	282(72.87)	38.434	0.000
IFG,n(*%*)	5(1.82)	13(4.71)	18(6.55)	30(11.11)	21.916	0.000	3(0.76)	12(3.00)	18(4.63)	52(13.44)	70.318	0.000
HC,n(*%*)	10(3.64)	10(3.62)	13(4.73)	22(8.15)	7.827	0.050	35(8.88)	44(11.00)	57(14.65)	68(17.57)	15.414	0.001
MetS,n(*%*)	5(1.82)	11(3.99)	28(10.18)	75(27.78)	116.677	0.000	20(5.08)	30(7.50)	55(14.14)	139(35.92)	175.572	0.000
Height(cm),M(P25,P75)	168(164,172)	170(165,172)	168(164,172)	170(165,172)	6.876	0.076	157(152,160)	156(153,160)	156(153,160)	157(154,160)	4.568	0.206
Weight(kg),M(P25,P75)	63(58.3,70)	65.8(60,70.8)	67.1(63,72.2)	70(65,75)	73.885	0.000	55(50,62)	58(52,63)	59(53,65)	60(55,65)	61.115	0.000
WC(cm),M(P25,P75)	82(78,87)	84(79,90)	85(80,91)	87(82,92)	51.074	0.000	80(73,85)	82(77,87)	82(76,88.5)	83(78,90)	46.229	0.000
BMI(kg/m^2^),M(P25,P75)	22.72(20.96,24.54)	23.13(21.46,25.06)	23.88(22.31,25.71)	24.27(22.86,26.15)	69.485	0.000	22.86(20.80,24.84)	23.44(21.70,25.56)	24.06(21.79,26.04)	24.44(22.81,26.37)	61.836	0.000
SBP(mmHg),M(P25,P75)	124(118,130)	126(120,130)	126(120,131)	125(120,131)	5.700	0.127	123(114.5,129.13)	122.5(115,130)	125(118,130)	125(120,131)	21.678	0.000
DBP(mmHg),M(P25,P75)	77.5(70,81)	76(71,80)	77.5(72.5,82)	77.5(72,81)	5.076	0.166	75(69.38,80)	75(69,80)	76(70,80)	76(71,80)	15.055	0.002
FPG(mmol/L),M(P25,P75)	4.53(4.15,4.98)	4.82(4.45,5.26)	5.07(4.62,5.46)	5.30(4.80,5.73)	162.144	0.000	4.67(4.32,5.04)	4.87(4.52,5.32)	5.01(4.65,5.39)	5.26(4.81,5.74)	187.618	0.000
TG(mmol/L),M(P25,P75)	0.6(0.5,0.7)	0.9(0.80,0.98)	1.2(1.10,1.36)	1.97(1.62,2.71)	948.688	0.000	0.7(0.6,0.8)	1.04(0.92,1.15)	1.40(1.30,1.59)	2.16(1.80,2.86)	1374.340	0.000
TC(mmol/L),M(P25,P75)	4.2(3.7,4.8)	4.49(3.98,5.18)	4.70(4.10,5.20)	4.80(4.20,5.42)	60.893	0.000	4.7(4.08,5.32)	5(4.4,5.6)	5.20(4.60,5.80)	5.20(4.70,6.00)	76.594	0.000
LDL-C(mmol/L),M(P25,P75)	2.56(2.04,3.14)	2.82(2.29,3.30)	2.87(2.40,3.33)	2.89(2.28,3.40)	21.846	0.000	2.77(2.12,3.4)	3.07(2.51,3.6)	3.11(2.57,3.82)	3.20(2.52,3.80)	37.410	0.000
HDL-C(mmol/L),M(P25,P75)	1.31(1.09,1.66)	1.29(1.03,1.62)	1.16(0.93,1.46)	1.16(0.90,1.44)	47.789	0.000	1.44(1.16,1.74)	1.31(1.12,1.67)	1.26(1.10,1.62)	1.17(0.90,1.51)	79.639	0.000
LAP,M(P25,P75)	9.72(7.05,14.4)	16.31(11.80,21.60)	24.70(19.50,32.40)	43.45(31.43,62.63)	606.600	0.000	14.31(9.80,19.43)	25.28(18.71,30.60)	34.00(24.73,43.20)	56.00(42.50,79.20)	922.382	0.000
WHtR,M(P25,P75)	0.49(0.46,0.52)	0.59(0.47,0.53)	0.51(0.48,0.55)	0.51(0.48,0.55)	40.259	0.000	0.51(0.47,0.54)	0.52(0.48,0.56)	0.52(0.48,0.56)	0.53(0.49,0.57)	38.789	0.000

### Logistic regression analysis for risk factors of PHT

**Fig. 3:**
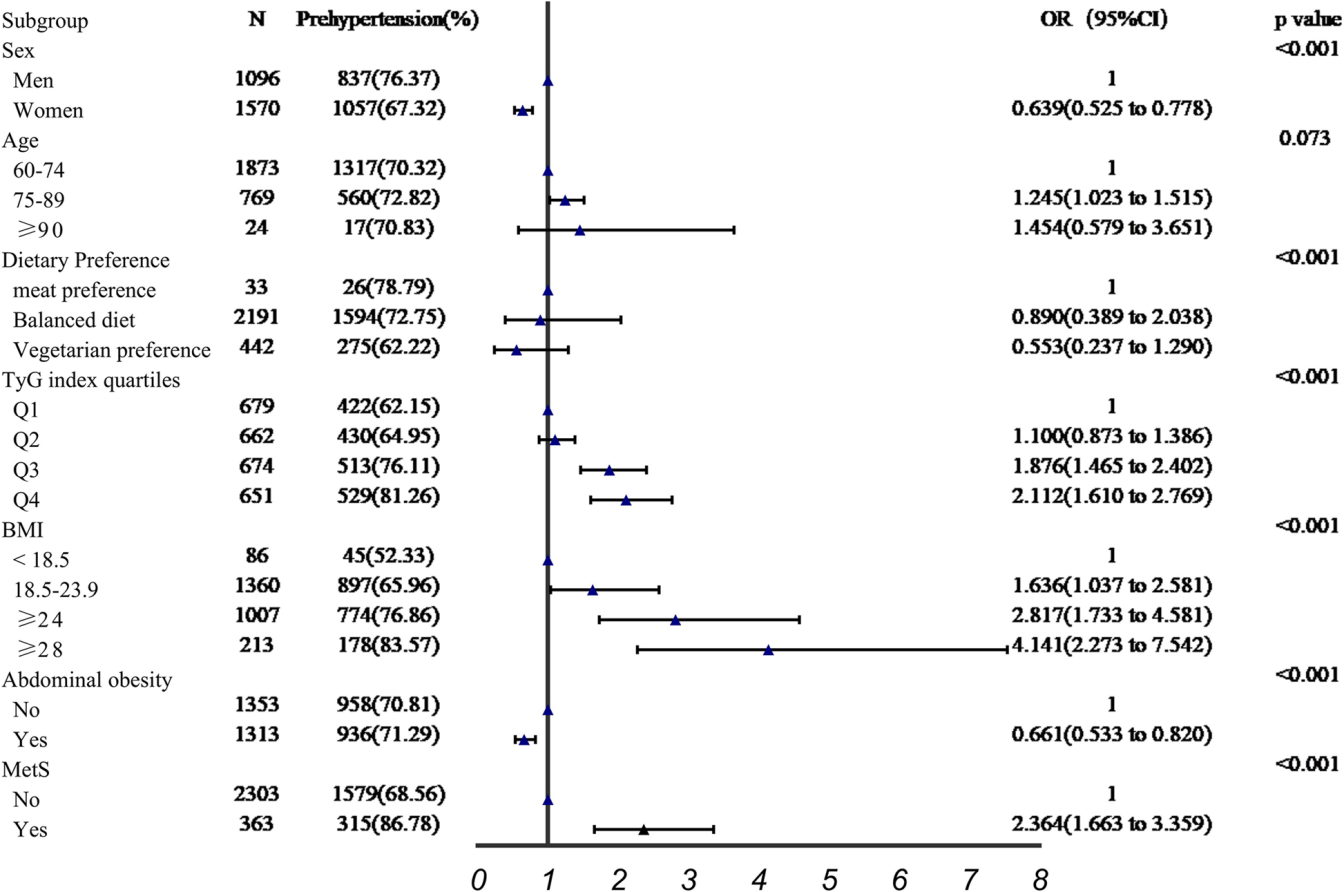
Binary logistic regression analysis of PHT

Figure 3 showed the results of the logistic regression model analysis for PHT risk factors. The findings demonstrated that the sex, dietary preference, TyG index quartiles, BMI levels, abdominal obesity and MetS were statistically significant in subjects with PHT and normotension. The TyG index in Q3 (1.876, 95%CI:1.465 to 2.402) and Q4(2.112, 95%CI: 1.610 to 2.769) group were statistically significant associated with PHT compared with Q1 group. Apart from that, participants with MetS was a risk factor for PHT (OR:2.364, 95%CI:1.663 to 3.359), but abdominal obesity was a protective factor for PHT(OR:0.661, 95%CI:0.533 to 0.820). The risk of PHT considerably increased as the BMI levels climbed (18.5–23.9: OR:1.636, 95%CI:1.037 to 2.581, ≥ 24: OR:2.817, 95%CI:1.733 to 4.581, ≥ 28: OR:4.141, 95%CI:2.273 to 7.542).

### Relationship between TyG index and prevalence of PHT

The relationship between TyG index quartiles and the risk of PHT prevalence in men and women were shown in Table 3. In this regression model, the Q1 quartile of TyG index served as the reference value (OR = 1). The risk of PHT was significantly higher in the high quartile group Q3 and Q4 of TyG index than that in the low quartile group Q1 in both males (Q3:2.72, 95%CI:1.81–4.09; Q4: 2.76, 95%CI: 1.82–4.19) and females (Q3:2.46, 95%CI: 1.80–3.35; Q4: 3.02, 95%CI: 2.18–4.18). The TyG index and the risk of PHT were still correlation significantly after adjusting confounding variables in multivariable model (model 1, model 2, model 3).

**Table 3:** Risk of prehypertension in the quartile of TyG index

Variables	PHT (n,*%*)	Unadjusted	Model 1	Model 2	Model 3
Men					
Q1(< 7.97)	177(64.36)	1.00(ref)	1.00(ref)	1.00(ref)	1.00(ref)
Q2(7.97–8.29)	205(74.28)	1.56(1.08–2.26)	1.57(1.08–2.29)	1.58(1.08–2.29)	1.57(1.06–2.30)
Q3(8.29–8.71)	229(83.27)	2.72(1.81–4.09)	2.69(1.78–4.06)	2.67(1.78–4.07)	2.69(1.73–4.18)
Q4(> 8.71)	226(83.70)	2.76(1.82–4.19)	2.78(1.82–4.25)	2.77(1.80–4.25)	2.83(1.77–4.54)
*P* for trend		< 0.001	< 0.001	< 0.001	< 0.001
Women					
Q1(< 8.12)	225(57.11)	1.00(ref)	1.00(ref)	1.00(ref)	1.00(ref)
Q2(8.12–8.48)	222(55.50)	0.92(0.69–1.22)	0.88(0.66–1.18)	0.87(0.65–1.16)	0.87(0.65–1.17)
Q3(8.48–8.82)	299(76.86)	2.46(1.80–3.35)	2.41(1.76–3.31)	2.34(1.70–3.21)	2.38(1.71–3.31)
Q4(> 8.82)	311(80.36)	3.02(2.18–4.18)	2.86(2.05–3.98)	2.64(1.88–3.70)	2.75(1.91–3.97)
*P* for trend		< 0.001	< 0.001	< 0.001	< 0.001

Unadjusted: Age, abdominal obesity, general obesity, TyG index

Model 1: Adjustment for Education level, Marital status, Physical activity, Dietary Preference, Current smoking and Current drinking

Model 2: The adjustment of IFG and HC were added on the basis of Model 1

Model 3: The adjustment of FPG, TC, HDL and LDL were added on the basis of Model 2

### Predictive value of TyG index and other common anthropometric indices/ratios of obesity on the risk of HTN

**Fig. 4:**
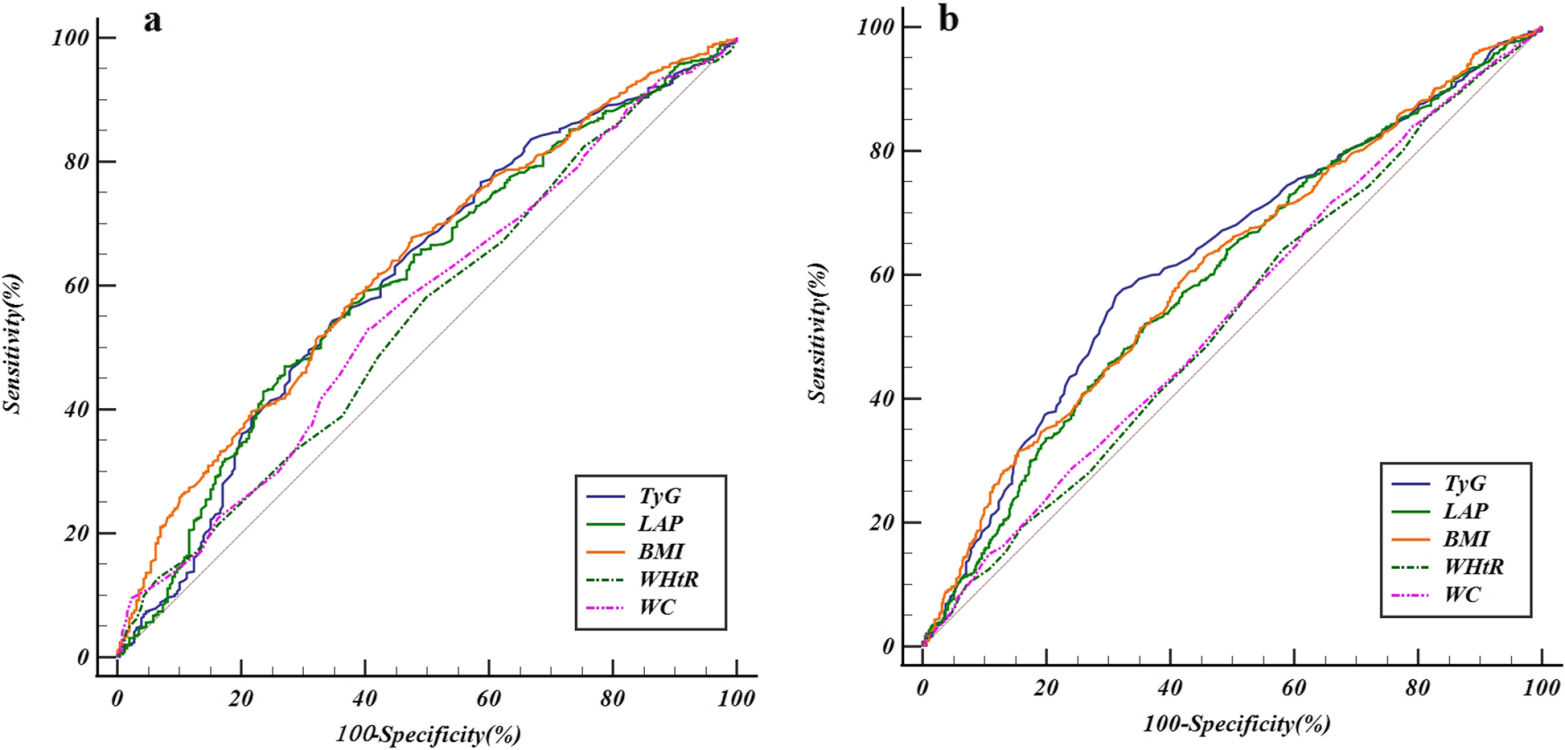
ROC curve of PHT in males and females. **a** Males. **b** Females

Table 4 and Fig. 4 showed the ROC curve analysis findings of PHT. The results comprised cut-off value, sensitivity, specificity, Youden index, and the AUC of TyG index and other obesity indicators. The AUC of these indexes were all greater than 50%, which was diagnostic significance for PHT. The cut-off value of TyG index was 8.29 for men and 8.50 for women. The TyG index of women has the greatest AUC value (0.626, 95%CI:0.602 to 0.650), followed by BMI(0.609, 95%CI:0.584 to 0.633). In contrast to women, the AUC of BMI (0.633, 95%CI:0.604 to 0.661) was higher in the participants of men than TyG (0.614, 95%CI:0.584 to 0.643) and LAP (0.611, 95%CI:0.581 to 0.640).

**Table 4:** ROC curve analysis of PHT in men and women

Variables	Cut-off value	Sensitivity (*%*)	Specificity (*%*)	Youden index	AUC(*95%CI*)	*Z*	*P*
Men							
TyG	8.29	54.36	65.25	0.196	0.614(0.584 to 0.643)	5.587	< 0.001
LAP	22.78	46.95	72.97	0.199	0.611(0.581 to 0.640)	5.546	< 0.001
BMI	22.72	67.74	52.51	0.202	0.633(0.604 to 0.661)	6.881	< 0.001
WHtR	0.49	58.06	50.19	0.082	0.549(0.519 to 0.579)	2.421	0.016
WC	84.50	53.05	59.46	0.125	0.563(0.533 to 0.593)	3.111	0.002
Women							
TyG	8.50	56.58	68.81	0.254	0.626(0.602 to 0.650)	8.514	< 0.001
LAP	29.43	52.03	64.13	0.162	0.597(0.572 to 0621)	6.408	< 0.001
BMI	23.42	60.55	56.92	0.175	0.609(0.584 to 0.633)	7.309	< 0.001
WHtR	0.50	64.05	41.91	0.059	0.527(0.502 to 0.552)	1.727	0.084
WC	77.00	71.81	33.92	0.057	0.536(0.511 to 0.561)	2.302	0.021

### Interaction between TyG index and obesity situation on PHT

Table 5 showed the results from the analysis of the interaction. The interaction was analyzed by the critical value of TyG index and obesity status. The cut-off point of the ROC curve in men > 8.29 and women > 8.5 was used to designate the TyG index as positive ( +). BMI ≥ 28 was defined as general obesity positive ( +), while abdominal obesity positive( +) was defined as having a waist circumference over 85 cm for women and 90 cm for men. Regarding males, PHT risk was 5.34 times higher in subgroups with high TyG indexes and general obesity than it was in subgroups with low TyG indexes and non-obesity (aOR: 5.34, 95%CI:1.86 to 15.30). AP was 0.87 (95%CI:0.72 to 1.02), indicating that 87% of PHT was caused by the combined interaction of both risk factors. SI was10.48 (95%CI:3.43 to 31.97), suggesting that this subgroup was 10.48 times higher than that of participants exposed to a single risk factor. The same applies to women (aOR:6.87, 95%CI:3.24 to 14.55), AP:0.89(95%CI:0.79 to 0.98), SI:12.46 (95%CI:5.61 to 27.69). Contrary to general obesity, individuals with high TyG index and non-abdominal obesity were more susceptible to developing PHT in both men(aOR:2.36, 95%CI:1.63 to 3.40; AP:0.60, 95%CI:0.38 to 0.83; SI:3.53, 95%CI:1.99 to 6.26) and women(aOR:3.03, 95%CI: 2.04 to 4.49; AP: 0.66, 95%CI: 0.51 to 0.82; SI: 3.89, 95%CI: 2.54 to 5.98).

**Table 5:** Interaction between TyG index and general obesity and abdominal obesity

Variable		*OR(95%CI)*	*aOR(95%CI)*	Measures of interaction	
				Multiplicative interaction	Additive interaction
Men					
TyG^a^	General obesity				
-	-	1	1	OR = 5.13(2.04–17.23) aOR = 5.33(2.09–18.13)	RERI = 25.01(-22.12 to 72.15) AP = 0.87(0.72 to 1.02) S = 10.48(3.43 to 31.97)
-	+	2.73(1.83 to 2.63)	2.43(0.70 to 8.45)		
+	-	2.17(1.62 to 2.92)	2.20(1.61 to 3.03)		
+	+	5.13(1.82 to 14.49)	5.34(1.86 to 15.30)		
TyG^a^	Abdominal obesity				
-	-	1	1	OR = 2.27(1.50–3.52) aOR = 2.34(1.50–3.72)	RERI = 3.81(-1.02 to 8.65) AP = 0.60(0.38 to 0.83) S = 3.53(1.99 to 6.26)
-	+	2.14(0.73 to 1.78)	1.15(0.73 to 1.80)		
+	-	2.31(1.63 to 3.28)	2.36(1.63 to 3.40)		
+	+	2.27(1.49 to 3.48)	2.34(1.49 to 3.67)		
Women					
TyG^a^	General obesity				
-	-	1	1	OR = 7.45(3.76 to 16.96) aOR = 6.87(3.44 to 15.74)	RERI = 23.75(-3.33 to 50.83) AP = 0.89(0.79 to 0.98) S = 12.46(5.61 to 27.69)
-	+	1.59(0.91 to 2.77)	1.54(0.88 to 2.71)		
+	-	2.73(2.17 to 3.44)	2.52(1.98 to 3.22)		
+	+	7.45(3.54 to 15.66)	6.87(3.24 to 14.55)		
TyG^a^	Abdominal obesity				
-	-	1	1	OR = 3.11(2.31 to 4.20) aOR = 2.76(2.02 to 3.80)	RERI = 6.22(-0.12 to 12.55) AP = 0.66(0.51 to 0.82) S = 3.89(2.54 to 5.98)
-	+	1.19(0.90 to 1.58)	1.12(0.84 to 1.49)		
+	-	3.35(2.29 to 4.92)	3.03(2.04 to 4.49)		
+	+	3.11(2.31 to 4.20)	2.76(2.01 to 3.79)		

*aOR* adjustment for education level, physical activity, dietary preference, current smoking, current drinking, marital status, age, FPG

^a^Grouped by the cut-off values in Table 4

## Discussion

Results of this cross-sectional study in relation to population research demonstrate that as the TyG index quartile group climbed, so increased the risk of PHT. The connection between PHT risk and TyG index remained significant after adjusting for confounding factors.

Insulin regulation and various bodily functions are both impacted by the IR mechanism of action. It is considered to be an unfavorable metabolic condition brought on by elevated triglyceride and insulin levels as a result of insulin insensitivity, one of the primary triggers to the dysfunction of lipid and glucose metabolism [23]. Under normal physiological circumstances, insulin widens blood vessels by boosting the synthesis of nitric oxide in vascular endothelial cells, facilitating the transfer of nutrients and controlling the dynamic balance of glucose in the body [24]. When the body is in a state of IR, the sensitivity of islets decreases, vasoconstrictor factors increase, pathological vascular sclerosis occurs and abnormal vasoconstriction takes place [25]. In addition, the renin–angiotensin–aldosterone system and sympathetic nervous system function are out of balance, which results in endothelial and smooth muscle cell appear hypertrophy, peripheral vascular resistance increases, water and sodium retention increased blood volume and ultimately elevated blood pressure [26, 27]. In cross-sectional studies, cohort studies and controlled trials, IR has been demonstrated to be one of the significant independent risk factors for various of chronic diseases [28–30]. The change in triglyceride level in the body is the another determining factor for the occurrence of IR [31]. Chronic oxidative stress creates greater challenges for liver and muscle tissue, when islet cells are exposed to an environment of high triglycerides [32]. In addition, the body is in a condition of obesity, excess free fatty acids in the liver or muscle tissue and other places appear abnormal accumulation. Obesity reduces the ability of adipose tissue to store fat and persistent proinflammatory response will be triggered that promotes IR develop [33]. IR worsens insulin sensitivity and promotes more lipid accumulation [34], toxic substances are created by ectopic fat accumulation and lipotoxicity damages organs [35].

IR can be effectively replaced by the TyG index, a biochemical index created by combining triglycerides and FPG [36]. A 2021 Korean cohort study showed a good causal relationship between TyG and IR [37]. Contrast with the hyperinsulinemic-euglycemic clamp (HIEC), the gold standard for IR diagnostics [38]. The operation of HIEC is intrusive and necessitates substantial financial and technical assistance, making it challenging to implement in clinical practice [39]. HOMA-IR is currently a popular alternative IR indication in clinical practice. Although the cost of measurement is less than that of HIEC, the collection of fasting plasma insulin data required for measurement has various issues, such as poor reuse rate and large error in the measurement process [40]. The results of Irace showed that TyG index is more strongly correlated with lipid Profile in the blood than homeostasis Model assessment of insulin resistance (HOMA-IR) [41]. According to Sarang Jeong's research findings in the Korean population, the TyG index is considered to be a more trustworthy indicator to replace IR than the HIEC and HOMA-IR [42]. TyG index is more suitable for extensive population investigation in the field of public health [43]. The TyG index has gained popularity in recent years for use in CVD prediction, including HTN. When the terms TyG index and CVD were searched on PubMed, the results revealed a total of 69 papers that were pertinent since 2014, including 12 in 2021, 30 in 2022, and 16 in 2023. Among them, there are 14 papers related to HTN. Those with PHT are more prone to develop HTN than those with normal blood pressure and they also have a greater prevalence of CVD [44, 45]. Yu Yan [46] and Zegui Huang [47] et al. published a study on the association between TyG index and HTN risk. The findings demonstrated that the increase in TyG index was independently correlated with the progression of arteriosclerosis and that a long-term increase in TyG index was linked to a higher chance of ischemic stroke. In addition, the prevalence of PHT was correlated with TyG index and increased with the increase of TyG index [14, 48]. In recent years, a large number of cohort studies have been conducted on the relationship between TyG index and hypertension and cardiovascular diseases, and the results show that there is a significant causal relationship between TyG index and hypertension, and the results of the measurement response relationship curve show that TyG index is positively correlated with the risk of hypertension [49, 50]. The difference is that the causal relationship between TyG and cardiovascular disease in people with prehypertension remains unclear. A prospective cohort study published in 2021 showed that TyG index was significantly correlated with the progression of arterial stiffness in people with hypertension. However, the causal relationship between TyG index and arterial stiffness in people with prehypertension cannot be fully confirmed [51].

The occurrence of HTN and diabetes has similar pathophysiological process and the synergistic effect exists between them [52]. People with one condition, high blood pressure or diabetes, are more likely to have the another [52]. Nearly two-thirds of diabetic individuals have HTN [53]. Therefore, the important influencing factors of PHT should be sought to predict its occurrence early. This is important for reducing the prevalence of HTN and diabetes, reducing the risk of CVD and cerebrovascular diseases, and reducing the global burden of disease.

In this study, elderly people with diabetes and HTN were excluded. To some extent, this can reduce the interaction and synergistic effect between HTN and diabetes. According to a report on the state of HTN in China that Zengwu Wang [15] published in 2018, PHT is more common than 55% among the elderly population 65 years of age and older and the prevalence grows with age. The health issues facing the elderly population are more pressing given the grim trend of global population aging.

ROC curve results of this cross-sectional study showed that TyG index was the best predictor of PHT in the elderly female population and its AUC value was the largest. Different from elderly women, the AUC of BMI in the ROC curve of elderly men was slightly greater than that of TyG. This results were matched thefinding of ZhenYu Zeng [54]. Through the analysis of the sensitivity and specificity of TyG index, among the male individuals, 65.25% of the participants may be accurately classified as being normotension (specificity = 65.25), while 54.36% of the participants can be classified correctly as PHT (sensitivity = 54.36). The Youden index of BMI in men was the best at identifying patients from non-patients. With a likelihood of 56.58% to identify actual patients (sensitivity = 56.58) and a probability of 68.81% to identify non-patients (specificity = 68.81), TyG had the highest Youden index among the female subjects. Additionally, TyG-BMI, TyG-WC, TyG-WHtR, and other Tyg-related indicators were developed and used in the study of metabolic syndrome by TaiwoHRaimi, MMirr, and other researchers [55, 56]. The findings of their study demonstrated that TyG-related indicators had a greater capacity to predict metabolic syndrome than the TyG index. A retrospective study was recently published in April 2023 to investigate the relationship between TyG correlation index (TyG-BMI) and hypertension and prehypertension. The results of this study showed that TyG-BMI was consistently positively correlated with hypertension and prehypertension. It is a reliable indicator to predict the occurrence of prehypertension and hypertension [57]. The application of the TyG-related index may help us to better predict the risk of PHT in future studies.

In addition, the study took into account the relationship between obesity and PHT. The results revealed that PHT risk and general obesity were positively correlated. This is in line with the findings of SA Isezuo, Abdellatif Moussouni, Hu L et al. [10, 58, 59]. Contrary to the results of other investigations, this study found a negative correlation between PHT risk and abdominal obesity as defined by waist circumference. The appearance may be affected by the selection of research objects. Those with diabetes and HTN were not included in this study because these conditions are known to have higher WC.

Some limitations in this study should be considered. First of all, this study is a cross-sectional one rather than a cohort study that takes into account the causal relationship between the risk of PHT and the TYG index.

In addition, a significant portion of elderly individuals with diabetes and HTN were excluded, which reduced the sample size. Finally, the relationship between WC and PHT is different from other studies and needs to be confirmed by further research. Notwithstanding these limitations, this study excluded two significant confounders, HTN and diabetes, which reduced their interactions and concerned the link between PHT and TyG index.

## Conclusions

In conclusion, the results of this study demonstrated that substantial correlation between TyG index and the prevalence of PHT in the elderly people without diabetic and HTN. TyG index was proved to be an effective predictor of the risk of PHT. Although the interaction between PHT and general obesity is confirmed in this study, the relationship with abdominal obesity still needs to be discussed. Cohort studies can be employed in further research to elucidate the mechanism between TyG index and PHT risk. Moreover, TyG index can be further coupled with various anthropometric indicators to discover a more accurate predictor of PHT in clinical work. This can achieve the early prevention of PHT and lower the mortality from chronic diseases.

## Data Availability

The datasets analyzed during the current study are not publicly available due to the personal privacy but are available from the corresponding author on reasonable request.

## Abbreviations

PHT: Prehypertension
HTN: Hypertension
CVD: Cardiovascular disease
TyG index: Triglyceride glucose index
IR: Insulin resistance
WC: Waist circumference
FPG: Fasting blood glucose
TC: Total cholesterol
TG: Triglycerides
HDL-C: High-density lipoprotein cholesterol
LDL-C: Low-density lipoprotein cholesterol
SBP: Systolic blood pressure
DBP: Diastolic blood pressure
BMI: Body mass index
HC: Hypercholesterolemia
IFG: Impaired fasting glucose
MetS: Metabolic syndrome
ROC: Receiver operating characteristic
AUC: Area under the curve
SD: Standard deviation
OR: Odds ratio
CI: Confidence interval
HOMA-IR: Model assessment of insulin resistance
HIEC: Hyperinsulinemic-euglycemic clamp
